# A spatio-temporal transcriptomic and proteomic dataset of developing *Brassica napus* seeds

**DOI:** 10.1038/s41597-025-05115-4

**Published:** 2025-05-07

**Authors:** Dominic Knoch, Nils Rugen, Johannes Thiel, Marc C. Heuermann, Markus Kuhlmann, Paride Rizzo, Rhonda C. Meyer, Steffen Wagner, Jos H. M. Schippers, Hans-Peter Braun, Thomas Altmann

**Affiliations:** 1 Leibniz Institute of Plant Genetics and Crop Plant Research (IPK), Department of Molecular Genetics, Corrensstraße 3, 06466 Seeland OT, Gatersleben, Germany; 2 Institute of Plant Genetics, Leibniz University Hannover, Herrenhäuser Straße 2, 30419 Hannover, Germany

**Keywords:** Seed development, Transcriptomics, Proteomics

## Abstract

Oilseed rape (*Brassica napus*) seeds are of major economic and nutritional value since they are rich in both oil and proteins, which accumulate predominantly in the embryonic cotyledons during the filling period. Developmental phases such as embryogenesis, seed filling, and maturation have been associated with specific changes in the transcriptional landscape and are controlled by interactions of regulatory components, particularly transcription factors and cis-regulatory elements. However, the global changes on the protein level remain largely elusive. Here, we investigated the dynamics of seed development by an integrative analysis of the seed transcriptome and proteome. Plants of the winter-type cultivar Express 617 were grown under controlled, field-like conditions in the IPK PhenoSphere, and developing seeds were collected for temporally and spatially resolved multi-omics analyses. The dataset covers five stages, from pre-storage to seed maturation, and includes spatial information on four dissected organs/tissues. It provides comprehensive insights into differentiation and developmental processes of the *Brassica napus* seed and may serve as starting point to select potentially important genes for detailed functional investigations.

## Background & Summary

Oilseed rape (*Brassica napus* L.), also known as rapeseed, is an oilseed crop with immense global importance. Besides the usage of its oil for human consumption and industrial applications, protein from rapeseed meal is deemed to play an increasing role in food and animal feed. Despite its global importance, the regulatory processes directing seed development, filling and maturation, and finally, the nutritional content, remain largely elusive. Seed development depends on the co-ordinated spatio-temporal expression of various genes involved in diverse processes, like cell division, differentiation, seed filling, desiccation, and finally seed dormancy. Different transcription factors are of critical importance, which control the expression levels and patterns of genes by transcriptional regulation^1^. The development of *Brassica napus* seeds also involves a complex interplay of genetic, epigenetic, developmental, and metabolic processes that are spatially and temporally regulated. The developmental transitions from seed morphogenesis to filling and maturation requires a reorganization of gene expression and changes in the underlying gene regulatory networks. Understanding these complex dynamics is crucial for improving seed traits such as oil and protein content, nutritional value, taste, and early seedling vigour.

Recent advancements in omics technologies, such as genomics, transcriptomics, proteomics, and metabolomics, provide unprecedented opportunities to gain insights into these biological processes. Seed development has been extensively studied in the closely related model plant *Arabidopsis thaliana*, in particular, on the histological, transcriptomic^1–6^, and proteomic level^7–9^. Global changes in gene expression and their coordination during different stages of embryo/seed development allow conclusions about the underlying genetic mechanisms and metabolic processes associated with the accumulation of storage compounds. Although related, *Brassica* seeds differ substantially from *Arabidopsis* seeds in both, size and their composition of storage reserves^10^. In oilseed rape, seed development has been addressed on the morpho-histological level, as well as on the transcriptomic level in several studies^11–15^, deducing underlying regulatory networks^16–18^. Other omics layers including metabolomics^19^, proteomics^20–22^ or epigenomics^23–25^ have been targeted in previous studies. However, investigations with temporal and spatial resolution and a comprehensive integrative profiling of multiple omics layers are lacking. These would enable the inference of mechanistic relations among the components involved in the processes from gene/genome to phene/phenome.

In this study, we present a comprehensive spatio-temporal multi-omics profiling dataset of developing *B. napus* seeds, utilizing state-of-the-art transcriptomics and proteomics analyses. Plants of the German high oil cultivar Express 617 (00-quality), which has been widely used in rapeseed breeding programs and as parent for the creation of genetic mapping populations, were grown under field-like, but highly controlled environmental conditions in the container-based system of the IPK PhenoSphere^26^. Seeds were sampled at five distinct stages of development and dissected into different maternal and embryonic organs/tissues, namely the seed coat (SC; including endosperm), the outer (OC) and inner cotyledons (IC), and the radicle (RA; radicle and hypocotyl). Aliquots of the obtained material were subjected to mRNA sequencing and mass spectrometry-based proteomics. This dataset captures the spatial distribution of gene expression and protein abundance at key developmental stages, offering a valuable resource for dissecting the molecular mechanisms underlying seed development.

## Methods

### Plant cultivation under controlled, field-like conditions in the IPK PhenoSphere

Plants of the winter-type oilseed rape cultivar Express 617 were grown in the container-based cultivation system of the IPK PhenoSphere^26^. The experiment was performed between September 2020 and July 2021, covering a full growth-season. The weather regime of this cultivation was based on twelve years of hourly records (between 2008 and 2019) from IPK’s weather station. It was designed to be as representative as possible of a typical season avoiding stressful weather extremes and thus was considered to be benign and therefore favourable for plant growth and seed formation. For this reference dataset, undisturbed seed development, seed filling, and maturation was intended to achieve high seed quality and vigour. The historical weather data were subdivided into weeks starting January 1^st^ and a partitioning around medoids (PAM) algorithm was used to sort the 84 days from a certain week of all 12 years into three clusters according to their hourly temperature profile. Thereby, three medoid days, representative for a ‘sunny’, a ‘cloudy’, and an ‘intermediate’ temperature profile were selected for each week. The PhenoSphere air conditioning system was programmed to recreate the hourly characteristics of those days in terms of air temperature and relative air humidity, and we adjusted the illumination for seasonal changes in daytime length. For each week, the illumination programs were adjusted to the times of sunrise and sunset, the general light intensity of a certain season (spring, summer, and autumn), and to the intensity and frequency of light fluctuations of the medoid days. ‘Cloudy’ days are characterized by low light intensity and 8 hours of light fluctuations, ‘sunny’ days by high light and 2 hours of fluctuations, and ‘intermediate’ days have similar light maxima as sunny days but with 5 hours of light fluctuations with stronger intensity variation (amplitude). The three week-specific medoid days were randomly distributed, proportionally to the number of days allocated to the respective cluster, throughout the course of a particular week. In this manner, a full representative growth-season (September to July) was approximated. Wind was simulated by fans in low position on both sides of the compartment. Detailed information on the weather regime, including hourly aggregated temperature, relative air humidity, and light intensity records are provided as ‘01_climate_and_sensor_data’ at figshare^27^.

Seeds were stratified for two days in Petri dishes on moist filter paper in the dark at 4 °C. Plants were sown and grown in containers with a sowing density of 48 plants/m^2^. Two seeds per position were sown and later reduced to one seedling to ensure uniform plant density. Containers were filled with 1 m^3^ soil, divided into layers simulating natural soil layers: top soil from the field, loam, coarse sand, and coarse gravel, and were equipped with soil sensors at three different depths. Containers were saturated with 80 L of water before planting. Plants were watered by an automatic drip irrigation system and 30 g of ammonium sulphate fertilizer (DOMOGRAN®, DOMO Caproleuna GmbH, Leuna, Germany), dissolved in 4 L of water, was added to each container before flowering. Between November 5^th^ 2020 and March 31^st^ 2021, containers were moved out to a rainout-shelter during the winter months for hibernation providing natural cold (including frost) temperatures, which could not be simulated in the PhenoSphere, but are required for vernalisation of winter-type cultivars to ensure normal development and flowering induction. The weather simulation and cultivation in the PhenoSphere was continued until June 11^th^ 2021 after which the containers had to be moved outside. At that day, the operation of the indoor cultivation system had to be stopped for technical reasons. As the indoor simulated weather program was fully aligned with the natural season and the outdoor conditions were favourable during that period, the plants in the containers then placed under the open sky were seamlessly exposed to similar benign conditions for the remaining 4 days until the final sampling (stage 5). We have also provided an overlay of the measured temperatures inside and outside the PhenoSphere with historical records, as well as a photo documenting the normal growth phenotypes of the plants after overwintering as ‘02_temperature_plots’ at figshare^27^.

### Sampling and dissection of developing seeds for multi-omics analyses

**Fig. 1:**
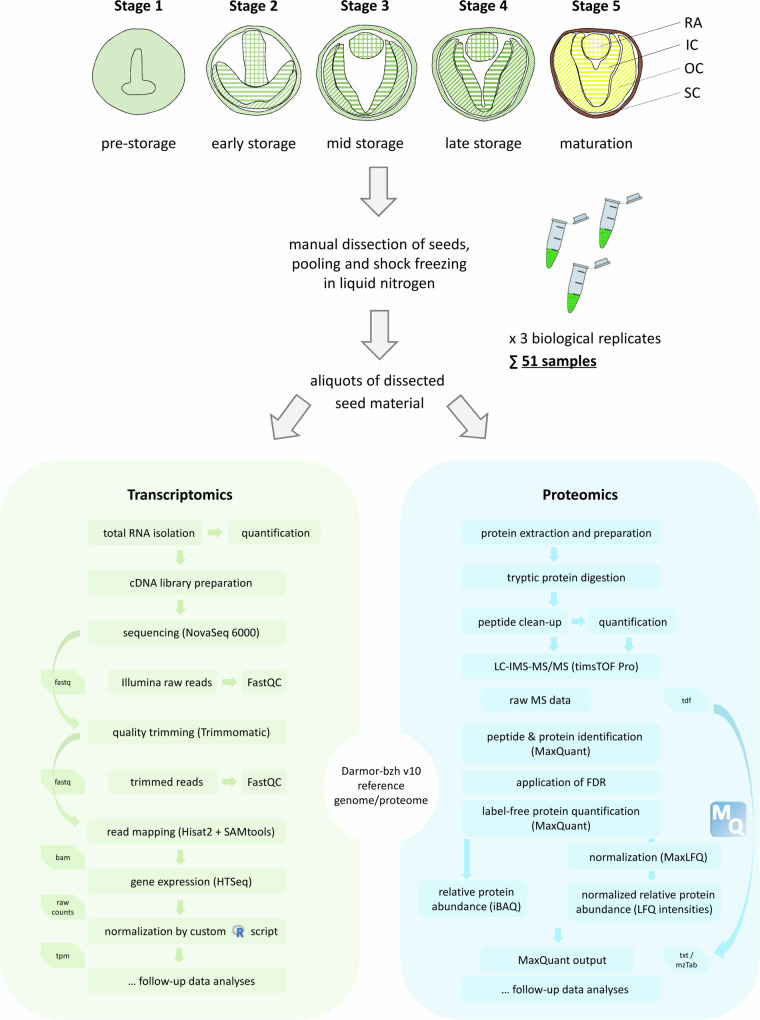
Overview of the collected samples and data generation. Flowcharts show an overview of sample collection, material pre-processing and the parallel pipeline for transcriptomics and proteomics data analyses steps and used software. A total of 51 samples were collected, comprising combinations of different organs/tissues and developmental stages. For stage 1, whole seeds were collected due to the small size of the embryo. Seeds of stages 2 to 5 were manually dissected. The abbreviations SC, OC, IC and RA refer to the seed coat, outer and inner cotyledons and the radicle, respectively. For further details, please see the Methods section.

Seeds were sampled at five key stages of development, previously defined by Borisjuk *et al*.^28^ (Fig. 1): The pre-storage phase (stage 1), which is characterized by cell division and expansion of the seed coat, alongside the transition of the embryo from the globular to bilateral symmetry. The early storage phase (stage 2), in which rapid changes in endosperm-embryo ratio occur driven by the growth of the embryo and expansion and bending of the radicle and both cotyledons. In this stage, all organs are photosynthetically active and transient storage processes culminate in the endosperm (soluble compounds, lipids) while starting in the embryo. The mid storage phase (stage 3), which is characterized by further expansion of the inner cotyledon, an exponential increase of embryo fresh weight, and lipid accumulation in the embryo axis and the outer cotyledon. This stage features the elimination of the endosperm, a slowing down of mitotic activity, gradual cessation of radicle growth, and dominance of cotyledons in size. Photosynthetic activity peaks in the outer cotyledon but decreases in the radicle. Additionally, pronounced storage parenchyma and the vascular network appear while embryo metabolism shifts from growth to storage. Strong gradients in starch, protein, and lipid deposition indicate spatial control of biosynthetic activity. The late storage phase (stage 4) is defined by a further increase of embryo fresh weight and predominant lipid accumulation in the embryo axis. At this stage, morphogenesis and tissue differentiation is completed, though cotyledon expansion continues. Storage product accumulation becomes dominant, with up to 50% of mRNA encoding storage proteins. Protein and lipid contents increase, along with progressive remobilization of the transient starch. The maturation phase (stage 5) is characterized by the maximum lipid content in inner and outer cotyledons. Embryo growth ceases, storage processes slow down and are eventually completed. Respiratory activity drops and controlled water loss (desiccation) leads to embryo shrinkage and a reduction in seed size.

Starting from the second stage, individual seeds were manually dissected into four organs/tissues: the seed coat (SC; testa including endosperm), the inner cotyledon (IC), the outer cotyledon (OC) and the radicle (RA; radicle and hypocotyl). Siliques and seeds were cooled on ice, and immediately after the dissection of an individual seed, the tissue was shock frozen in liquid nitrogen. For each of the 17 stage and organ combinations, three biological replicates were processed. Each replicate consisted of pooled material from seeds of multiple plants of a certain container (each replicate from a different container), resulting in a total of 51 samples (Table 1). To provide sufficient material needed for all omics analyses, between 250 and 500 individual seeds were dissected per replicate and stage. Sample tubes were cooled in liquid nitrogen and deep frozen plant material was homogenised 6x for 1.5 min at 30 Hz using a mixer mill (Retsch GmbH, Haan, Germany) and two 3 mm steel balls. Subsequently, aliquots of plant material were taken for RNA-sequencing and mass spectrometry–based proteomics.

**Table 1:** List of samples and metadata.

ID	Sample name ^†^	Development stage	Organ/Tissue	Rep.	BioSamples ID
1	L401HPKH21P01S1WS	stage 1 (pre-storage)	whole seed	1	SAMEA115475485
2	L401HPKH21P02S1WS	stage 1 (pre-storage)	whole seed	2	SAMEA115475486
3	L401HPKH21P03S1WS	stage 1 (pre-storage)	whole seed	3	SAMEA115475487
4	L401HPKH21P01S2SC	stage 2 (early storage)	seed coat	1	SAMEA115475488
5	L401HPKH21P02S2SC	stage 2 (early storage)	seed coat	2	SAMEA115475489
6	L401HPKH21P03S2SC	stage 2 (early storage)	seed coat	3	SAMEA115475490
7	L401HPKH21P01S2IC	stage 2 (early storage)	inner cotyledon	1	SAMEA115475491
8	L401HPKH21P02S2IC	stage 2 (early storage)	inner cotyledon	2	SAMEA115475492
9	L401HPKH21P03S2IC	stage 2 (early storage)	inner cotyledon	3	SAMEA115475493
10	L401HPKH21P01S2OC	stage 2 (early storage)	outer cotyledon	1	SAMEA115475494
11	L401HPKH21P02S2OC	stage 2 (early storage)	outer cotyledon	2	SAMEA115475495
12	L401HPKH21P03S2OC	stage 2 (early storage)	outer cotyledon	3	SAMEA115475496
13	L401HPKH21P01S2RA	stage 2 (early storage)	radicle	1	SAMEA115475497
14	L401HPKH21P02S2RA	stage 2 (early storage)	radicle	2	SAMEA115475498
15	L401HPKH21P03S2RA	stage 2 (early storage)	radicle	3	SAMEA115475499
16	L401HPKH21P01S3SC	stage 3 (mid storage)	seed coat	1	SAMEA115475500
17	L401HPKH21P02S3SC	stage 3 (mid storage)	seed coat	2	SAMEA115475501
18	L401HPKH21P03S3SC	stage 3 (mid storage)	seed coat	3	SAMEA115475502
19	L401HPKH21P01S3IC	stage 3 (mid storage)	inner cotyledon	1	SAMEA115475503
20	L401HPKH21P02S3IC	stage 3 (mid storage)	inner cotyledon	2	SAMEA115475504
21	L401HPKH21P03S3IC	stage 3 (mid storage)	inner cotyledon	3	SAMEA115475505
22	L401HPKH21P01S3OC	stage 3 (mid storage)	outer cotyledon	1	SAMEA115475506
23	L401HPKH21P02S3OC	stage 3 (mid storage)	outer cotyledon	2	SAMEA115475507
24	L401HPKH21P03S3OC	stage 3 (mid storage)	outer cotyledon	3	SAMEA115475508
25	L401HPKH21P01S3RA	stage 3 (mid storage)	radicle	1	SAMEA115475509
26	L401HPKH21P02S3RA	stage 3 (mid storage)	radicle	2	SAMEA115475510
27	L401HPKH21P03S3RA	stage 3 (mid storage)	radicle	3	SAMEA115475511
28	L401HPKH21P01S4SC	stage 4 (late storage)	seed coat	1	SAMEA115475512
29	L401HPKH21P02S4SC	stage 4 (late storage)	seed coat	2	SAMEA115475513
30	L401HPKH21P03S4SC	stage 4 (late storage)	seed coat	3	SAMEA115475514
31	L401HPKH21P01S4IC	stage 4 (late storage)	inner cotyledon	1	SAMEA115475515
32	L401HPKH21P02S4IC	stage 4 (late storage)	inner cotyledon	2	SAMEA115475516
33	L401HPKH21P03S4IC	stage 4 (late storage)	inner cotyledon	3	SAMEA115475517
34	L401HPKH21P01S4OC	stage 4 (late storage)	outer cotyledon	1	SAMEA115475518
35	L401HPKH21P02S4OC	stage 4 (late storage)	outer cotyledon	2	SAMEA115475519
36	L401HPKH21P03S4OC	stage 4 (late storage)	outer cotyledon	3	SAMEA115475520
37	L401HPKH21P01S4RA	stage 4 (late storage)	radicle	1	SAMEA115475521
38	L401HPKH21P02S4RA	stage 4 (late storage)	radicle	2	SAMEA115475522
39	L401HPKH21P03S4RA	stage 4 (late storage)	radicle	3	SAMEA115475523
40	L401HPKH21P01S5SC	stage 5 (maturation)	seed coat	1	SAMEA115475524
41	L401HPKH21P02S5SC	stage 5 (maturation)	seed coat	2	SAMEA115475525
42	L401HPKH21P03S5SC	stage 5 (maturation)	seed coat	3	SAMEA115475526
43	L401HPKH21P01S5IC	stage 5 (maturation)	inner cotyledon	1	SAMEA115475527
44	L401HPKH21P02S5IC	stage 5 (maturation)	inner cotyledon	2	SAMEA115475528
45	L401HPKH21P03S5IC	stage 5 (maturation)	inner cotyledon	3	SAMEA115475529
46	L401HPKH21P01S5OC	stage 5 (maturation)	outer cotyledon	1	SAMEA115475530
47	L401HPKH21P02S5OC	stage 5 (maturation)	outer cotyledon	2	SAMEA115475531
48	L401HPKH21P03S5OC	stage 5 (maturation)	outer cotyledon	3	SAMEA115475532
49	L401HPKH21P01S5RA	stage 5 (maturation)	radicle	1	SAMEA115475533
50	L401HPKH21P02S5RA	stage 5 (maturation)	radicle	2	SAMEA115475534
51	L401HPKH21P03S5RA	stage 5 (maturation)	radicle	3	SAMEA115475535

^†^sample names represent a linear combination of the genotype code, the experiment, the pool/replicate, developmental stage and organ/tissue; e.g. ‘L401HPKH21P01S1WS’ = L401 (project internal code for Express 617) + HPKH21 (PhenoSphere experiment in 2020 to 2021) + P01 (first pool | biological replicate) + S1 (first developmental stage; pre-storage) + WS (whole seed).

### RNA-sequencing and transcriptome analysis

Total RNA was isolated from each sample (5–10 mg aliquots of pooled seed tissue) using the Absolutely RNA Microprep Kit (Agilent Technologies, Santa Clara, California, U.S.) according to the manufacturer’s protocol with minor modifications: the volume of the lysis buffer was scaled up to 200 µl per sample and supplemented with 1.5% polyvinylpolypyrrolidone 25 (PVP particle size 25000, Serva, Heidelberg, Germany) to remove polysaccharides, proteins and polyphenol compounds, which accumulate particularly in the seed coat. After homogenizing sample material in the lysis buffer by vortexing, samples were incubated for 20 minutes at 50 °C and subsequently centrifuged at 10,000 g for 2 min to separate the debris and PVP from the cell extract. The supernatant was transferred to a new 1.5 ml Eppendorf tube, mixed with an equal volume of 70% ethanol and immediately transferred to an RNA binding column. During further processing, DNA was removed by on-column DNase I treatment after the first washing-step. Purified RNA was eluted in 20 µl nuclease-free water and RNA quantity and purity were assessed using a NanoDrop spectrophotometer (Thermo Fisher Scientific, Bremen, Germany) and a Qubit^Tm^ 4 fluorometer (Thermo Fisher Scientific, Bremen, Germany). RNA was diluted to 50 ng/µl for generation of cDNA libraries. Libraries were constructed using the Illumina Stranded RNA Prep Kit (Illumina, Berlin, Germany). RNA-Sequencing was performed on a NovaSeq 6000 platform using a S2_XP v1.5 flow cell (Illumina, Berlin, Germany) and generated on average 61 million 109 bp paired-end (PE) sequencing reads per sample. Raw reads were trimmed using Trimmomatic (version 0.36)^29^ using the following setting: PE, LEADING:20, TRAILING:20, SLIDINGWINDOW:4:20 and MINLEN:50. Quality trimmed read pairs were mapped to the improved Darmor-*bzh* v10 reference genome^30^ using Hisat2 (version 2.1.0)^31^ with default parameters, and resulting BAM files were sorted and indexed using SAMtools (version 1.6)^32^. Subsequently, features were counted using HTSeq (version 1.99.2)^33^ with the arguments: htseq-count -f bam--max-reads-in-buffer = 100000000 -r pos -t CDS -i Parent, and normalised for sequencing depths and transcript length using the ‘tpm’ procedure^34^.

### Protein extraction, digestion and peptide clean-up

Proteins were extracted from 20 mg aliquots of the same dissected seed material used for the transcriptome analyses (aliquots of the same pools of sampled material). Proteins were prepared for mass spectrometry analysis via the single-pot-solid-phase-enhanced sample preparation (SP3) protocol developed by Hughes *et al*.^35^. We used a protocol from Mikulášek *et al*.^36^ with minor adaptations: 500 µl of 1x SDT buffer (4% [w/v] sodium dodecyl sulfate (SDS), 0.1 M dithiothreitol (DTT), 0.1 M Tris-HCL, pH 7.6) were added to each aliquot and incubated on a thermal shaker (TS-100, Kisker Biotech, Steinfurt, Germany) for 1 h at 60 °C and 1,000 rpm. After centrifugation for 10 min at 20,000 g the supernatant was transferred into a new reaction tube and sonicated in a water bath for 10 minutes (Elmasonic S30, Elma, Singen, Germany) and centrifuged again for 10 min at 20,000 g. From the supernatant, 30 µl were transferred into a new reaction tube and proteins were alkylated via incubation in 20 mM Iodoacetamide for 30 min at 600 rpm at room temperature in the dark. Alkylation was stopped by addition of 5 mM DTT. Sera-Mag™ magnetic carboxylate modified hydrophilic particles (GE Life Sciences, Chicago, Illinois, U.S.) were combined 1:1 with hydrophobic particles, and a total amount of 600 µg beads was added to each sample. Protein binding to the beads was induced by addition of 70 µl ethanol (100%) and subsequent incubation for 10 minutes at 1,000 rpm at 24 °C. Beads were pelleted on a magnetic rack for two minutes and proteins were washed 3 times with 140 µl of fresh 80% ethanol. After protein clean-up, beads were transferred in fresh 80% ethanol into low protein-binding tubes (Low Binding Micro Tubes, Sarstedt, Nümbrecht, Germany) and all ethanol was removed on the magnetic racks. Proteins were digested with 2 µg of sequencing grade modified Trypsin (V511A; Promega, Fitchburg, Wisconsin, U.S.) in 50 mM ammonium bicarbonate at 37 °C at 1,000 rpm overnight in a total reaction volume of 60 µl. Protease was activated prior to digestion following the manufacturer’s instructions. Digestion was stopped the next day and the pH of each sample adjusted to <3 by adding 1% (v/v) formic acid (FA).

Peptides were cleaned up via solid-phase extraction on SepPak Vac 1cc (50 mg) tC18 cartridges (Waters, Eschborn, Germany). Cartridges were wetted with 1 ml 100% acetonitrile and 1 ml 0.1% (v/v) formic acid in 50% (v/v) acetonitrile. Cartridge equilibration was performed by adding 2 × 1 ml of 0.1% FA (v/v) in H_2_O. Acidified peptides (pH < 3) were loaded onto the cartridges and washed 2 times with 0.1% FA (v/v) in H_2_O and eluted 2x in 200 µl of 0.1% FA (v/v) in 50% (v/v) acetonitrile. Cleaned peptides were dried in a vacuum centrifuge and stored at −20 °C. The final peptide concentration was determined with the Pierce™ peptide quantification kit (Thermo Fisher Scientific, Bremen, Germany) following manufacturers’ instructions.

### LC-IMS-MS/MS analysis

All samples were randomized prior to the LC-IMS-MS/MS analysis. The order of the samples can be found as ‘03_order_of_injections’ at figshare^27^. A nanoElute2 HPLC (Bruker Daltonics, Bremen, Germany) was coupled to a timsTOF Pro ion-mobility spectrometry quadrupole time-of-flight mass spectrometer (Bruker, Billerica, Massachusetts, U.S.). Peptides were reconstituted in 0.1% FA, and 200 ng peptides per sample were directly transferred onto an ‘Aurora ULTIMATE’ reversed-phase analytical column with integrated emitter tip (AUR3-25075C18-CSI; 25 cm × 75 µm inner diameter, IonOpticks, Fitzroy, Australia). Peptides were separated on the analytical column at 50 °C via a 70 min gradient (solvent A: 0.1% FA; solvent B: 0.1% FA in 100% acetonitrile) at a flow rate of 300 nl min^−1^. A linear gradient from 2% to 37% B for the first 60 min was followed by a 10 min washing step at 95% B. The timsTOF Pro mass spectrometer was operated in DDA PASEF mode, and the pre-installed method ‘DDA PASEF-standard_1.1sec_ cycletime’ was used. Automatic recalibration of ion mobility before each sample run was activated. MS and MS/MS scan range was 100 – 1,700 m/z, the ion mobility range (1/K0) was 0.6–1.6 V s^−1^ cm^−2^. A polygon filtering was applied in the m/z and ion mobility area to exclude the low m/z of singly charged ions for PASEF precursor selection. Ramp and accumulation time were set to 100 ms to achieve close to 100% duty cycle. The number of PASEF ramps was set to 10 with a charge maximum of 5. The quadrupole isolation width was set to 2 for m/z = 700 and 3 for m/z = 800. Collision energy was 20 eV for ion mobility (1/K0) 0.6 V s^−1^ cm^−2^ and 59 eV for ion mobility (1/K0) 1.6 V s^−1^ cm^−2^, respectively.

### Protein identification and quantification

MaxQuant 2.0.3.0^37,38^ was used to query acquired MS/MS spectra against the Darmor-*bzh* v10 reference protein sequence database. Carbamidomethyl (C) was specified as a fixed modification, oxidation (M) and acetylation (protein N-Term) were considered as variable modifications. Trypsin/P was specified as the proteolytic enzyme with 2 maximum missed cleavages. The minimal peptide length was set to 7 amino acids and the maximum peptide mass to 4,600 Da. Identification transfer between individual runs via the ‘Match between runs’ feature was enabled. The match time window was set to 0.7 min and the alignment time window to 20 min. The match ion mobility window was set to 0.05 and alignment ion mobility was set to 1. For all samples, the FDR was 1% at both the peptide spectrum match (PSM) and protein level. Calculation of ‘Intensity Based Absolute Quantification’ (iBAQ)^39^ values was activated, the options ‘Log fit’ and ‘charge normalization’ were enabled. Additionally, protein quantification and protein abundance normalization via the MaxLFQ algorithm^40^ was performed with a LFQ min. ratio count of 2. The ‘Stabilize large LFQ ratios’ and ‘Require MS/MS for LFQ comparisons’ were enabled. For follow-up analyses, both LFQ intensities and iBAQ values were reported. We use iBAQs as proxies to determine the detection of a protein in each sample, while utilizing the stricter and more normalized LFQ intensities for their relative quantification across samples. Prior to further analysis, MaxQuant output tables were filtered for non-plant contaminants, reversed sequences and proteins which were only identified based on modified peptides.

The HeLa quality control (QC) runs were analyzed using MaxQuant (version 2.6.7.0). The human protein sequences were downloaded from UniProt. The same search parameters as for the 51 rapeseed sample files were used with the following exceptions: In the settings for the calculation of iBAQ, the ‘log fit’ option was disabled. The ‘match between runs’ feature was disabled. Information regarding the mass accuracy of individual measurements, as well as most other QC parameters such as the number of identified MS/MS spectra was extracted from the ‘evidence.txt’ or the ‘summary.txt’ files generated by MaxQuant. Skyline version 24.1.0.414^41^ was used to calculate the mean chromatographic peak width and the total TIC area of each LC-MS run. QC Data were analyzed and visualized using Instant Clue^42^.

### Statistical and multivariate analyses

Statistical analyses, including the calculation of Pearson correlations between biological replicates, and data visualisation were performed in R (version 4.3.1; www.r-project.org) using RStudio (https://posit.co/download/rstudio-desktop/) under Microsoft Windows 10. Low-expressed genes with less than 10 raw counts and less than 1 tpm in two of the tree replicates in all statistical groups (combination of organ/tissue and developmental stage) were removed as they tend to reflect noise. Proteomics data (iBAQ values) were log_2_ transformed after adding a pseudo count of 1e-05. Both datasets were centred and scaled (z-scores) prior to principal component analysis (PCA). PCA was performed using the ‘pcaMethods’ R package^43^. UpSet plots were generated using the ‘UpSetR’ R package^44^. Data quality of raw and trimmed RNA-Seq reads was analysed using FastQC^45^ and MultiQC^46^. Q20 and Q30 values reported in Table 3 were obtained using the tool fastp^47^. Pairwise Pearson correlations between all samples were calculated using the filtered and log_2_ transformed tpm values and log_2_ transformed LFQ intensities, respectively. Correlation matrices were subsequently converted to distance matrices and a hierarchical cluster analysis was performed using the hclust function of the ‘stats’ R package (method = “average”). Data were visualized using the ‘pheatmap’ R package.

**Table 2:** Number of quantified protein groups and transcripts.

Sample set	Protein groups ^†#^		Peptides ^#^	Transcripts ^*^
	(all)	(unambiguous)		
L401HPKH21PS1WS	11,515	5,574	70,135	45,300
L401HPKH21PS2SC	11,321	5,479	68,140	44,134
L401HPKH21PS2IC	11,252	5,438	65,278	39,108
L401HPKH21PS2OC	11,256	5,457	65,507	38,385
L401HPKH21PS2RA	11,278	5,490	66,664	38,919
L401HPKH21PS3SC	11,366	5,519	69,897	42,910
L401HPKH21PS3IC	11,211	5,417	65,373	35,827
L401HPKH21PS3OC	11,157	5,397	64,699	35,298
L401HPKH21PS3RA	11,010	5,325	60,544	32,285
L401HPKH21PS4SC	11,043	5,347	64,106	42,201
L401HPKH21PS4IC	10,933	5,339	61,570	28,694
L401HPKH21PS4OC	10,649	5,196	57,929	36,603
L401HPKH21PS4RA	10,364	5,072	55,638	27,681
L401HPKH21PS5SC	10,240	4,968	53,411	35,375
L401HPKH21PS5IC	9,722	4,719	47,095	34,245
L401HPKH21PS5OC	9,521	4,664	47,566	32,567
L401HPKH21PS5RA	9,768	4,781	49,392	35,646

^†^ Filtered for non-plant contaminants and reversed sequences and proteins only identified based on modified peptides; iBAQ > 0.

^#^ quantified in at least one of three biological replicates.

^*^ Filtered for ≥ 10 raw counts & ≥1 tpm in at least two of three biological replicates.

**Table 3:** RNA-seq data quality and alignment rates.

Sample name	Raw reads	Raw Q20	Raw Q30	Cleaned reads	Effective rate	Alignment rate
L401HPKH21P01S1WS^†^	127,859,732	96.86%	92.41%	119,580,952	93.53%	96.67%
L401HPKH21P02S1WS^†^	115,307,254	97.09%	92.77%	109,225,622	94.73%	96.69%
L401HPKH21P03S1WS	34,203,156	89.93%	79.49%	26,493,542	77.46%	92.03%
L401HPKH21P01S2SC	61,681,776	92.25%	83.84%	50,853,760	82.45%	94.50%
L401HPKH21P02S2SC^†^	153,864,620	96.95%	91.76%	145,965,068	94.87%	95.58%
L401HPKH21P03S2SC	51,335,264	92.07%	83.32%	43,062,012	83.88%	94.58%
L401HPKH21P01S2IC	59,737,036	92.51%	84.43%	49,769,026	83.31%	94.54%
L401HPKH21P02S2IC	55,436,008	92.15%	83.60%	45,450,832	81.99%	93.94%
L401HPKH21P03S2IC	56,598,180	92.05%	83.30%	47,427,006	83.8%	94.12%
L401HPKH21P01S2OC	52,582,376	87.16%	75.71%	33,075,958	62.9%	92.71%
L401HPKH21P02S2OC	55,324,816	91.62%	83.00%	44,233,360	79.95%	94.07%
L401HPKH21P03S2OC	47,750,716	91.86%	83.08%	39,966,958	83.7%	94.14%
L401HPKH21P01S2RA	75,877,778	91.56%	82.89%	60,941,000	80.31%	94.19%
L401HPKH21P02S2RA	58,922,202	92.22%	83.81%	48,706,358	82.66%	94.20%
L401HPKH21P03S2RA	61,037,340	92.58%	84.28%	51,520,010	84.41%	94.43%
L401HPKH21P01S3SC	58,520,622	92.40%	84.00%	48,625,160	83.09%	94.55%
L401HPKH21P02S3SC	41,536,840	89.60%	78.66%	31,778,906	76.51%	94.12%
L401HPKH21P03S3SC	70,902,202	92.36%	84.00%	58,704,136	82.8%	94.69%
L401HPKH21P01S3IC	57,174,530	92.74%	84.60%	48,488,532	84.81%	94.50%
L401HPKH21P02S3IC	56,925,866	92.73%	84.49%	48,352,686	84.94%	91.29%
L401HPKH21P03S3IC	61,852,710	92.21%	83.92%	50,832,806	82.18%	94.24%
L401HPKH21P01S3OC	56,343,770	91.97%	83.75%	45,944,900	81.54%	94.59%
L401HPKH21P02S3OC	61,988,134	92.31%	83.99%	51,104,056	82.44%	94.90%
L401HPKH21P03S3OC	55,190,734	91.75%	83.23%	45,112,476	81.74%	94.16%
L401HPKH21P01S3RA	58,210,978	92.35%	84.04%	48,665,620	83.6%	90.22%
L401HPKH21P02S3RA	45,076,102	91.36%	81.90%	36,802,118	81.64%	93.90%
L401HPKH21P03S3RA	45,083,748	91.90 &	83.57%	37,643,966	83.5%	91.99%
L401HPKH21P01S4SC	69,110,944	92.21%	83.61%	56,955,304	82.41%	94.63%
L401HPKH21P02S4SC	62,347,942	92.08%	83.31%	51,496,366	82.6%	94.67%
L401HPKH21P03S4SC	38,070,432	90.61%	81.25%	30,124,978	79.13%	62.01%
L401HPKH21P01S4IC	59,763,534	91.69%	83.33%	48,209,358	80.67%	89.34%
L401HPKH21P02S4IC	57,901,048	91.57%	83.10%	46,983,040	81.14%	91.15%
L401HPKH21P03S4IC	59,675,370	91.92%	83.63%	49,164,688	82.39%	91.21%
L401HPKH21P01S4OC	54,616,104	92.24%	83.59%	45,588,602	83.47%	95.14%
L401HPKH21P02S4OC	86,928,382	92.39%	83.87%	72,064,632	82.9%	95.24%
L401HPKH21P03S4OC	45,334,990	92.16%	83.36%	38,134,180	84.12%	94.34%
L401HPKH21P01S4RA	38,028,528	88.53%	76.82%	27,555,172	72.46%	94.21%
L401HPKH21P02S4RA	43,192,266	90.78%	82.40%	34,062,326	78.86%	81.03%
L401HPKH21P03S4RA	26,717,546	91.67%	83.89%	21,620,578	80.92%	88.42%
L401HPKH21P01S5SC	53,958,852	90.21%	80.28%	40,904,514	75.81%	87.67%
L401HPKH21P02S5SC	47,935,620	91.88%	83.17%	39,275,238	81.93%	93.28%
L401HPKH21P03S5SC	52,952,590	89.71%	79.10%	40,678,310	76.82%	92.98%
L401HPKH21P01S5IC^†^	139,384,274	96.99%	92.59%	131,256,132	94.17%	95.59%
L401HPKH21P02S5IC	29,601,322	90.70%	80.78%	23,402,560	79.06%	84.44%
L401HPKH21P03S5IC	48,210,512	91.73%	83.48%	39,261,794	81.44%	92.47%
L401HPKH21P01S5OC^†^	125,696,100	97.24%	93.12%	118,913,304	94.6%	95.68%
L401HPKH21P02S5OC	56,230,528	92.06%	83.54%	46,615,812	82.9%	92.72%
L401HPKH21P03S5OC	51,183,090	92.27%	83.88%	42,169,884	82.39%	93.64%
L401HPKH21P01S5RA	44,607,826	91.70%	83.27%	36,586,086	82.02%	91.46%
L401HPKH21P02S5RA	46,079,518	92.23%	83.77%	38,518,470	83.59%	90.91%
L401HPKH21P03S5RA	41,519,114	92.02%	83.48%	34,307,982	82.63%	91.51%

^†^These five samples were repeated on a separate lane / sequencing run due to issues with library preparation.

## Data Records

A project description for the AVATARS project has been generated at BioStudies (EMBL-EBI) under the accession ‘S-BSST1715’ and sample meta information for all 51 samples has been deposited at BioSamples (see Table 1). In total, two comprehensive and fully congruent omics-datasets were generated. The mRNA-seq dataset reported in this paper has been deposited at ArrayExpress (E-MTAB-14603)^48^ and raw sequence reads were brokered to the European Nucleotide Archive (ENA)^49^. Both, raw counts and the normalized tpm values are available at ArrayExpress. The proteome data, including raw mass spectrometric data and the MaxQuant output files were submitted to the ProteomeXchange Consortium (https://proteomecentral.proteomexchange.org/) via the PRIDE^50^ partner repository with the dataset identifier ‘PXD053532’^51^.

## Technical Validation

### Experimental design

To further deepen the understanding of biological pathways and the roles of individual genes during seed development, it is crucial to have high-quality data and a good coverage of the transcriptome and proteome. The material for the analyses was obtained from oilseed rape (*Brassica napus* L.) plants of the high oil cultivar Express 617 (00-quality), grown in the controlled environment of the IPK PhenoSphere^26^. The plants were grown under natural, field-like, but highly controlled and reproducible environmental conditions simulating a typical growth season. For the gene expression and proteomic profiling, we placed a focus on four organs, the seed coat (SC), the inner cotyledon (IC), the outer cotyledon (OC), and the radicle (RA), and collected samples at five pre-defined stages of rapeseed seed development spanning the phases from pre-storage to seed maturation (Fig. 1). While the first stage was analysed as whole seeds due to the small size of the embryo proper, the later four stages were dissected into four different organs/tissues. To cover all 17 stage and organ/tissue combinations of the developmental series, we performed mRNA sequencing and a proteomics experiment using LC-IMS-MS/MS to obtain deep and consistent proteome coverage across samples. For the proteomics analyses, we used aliquots of the pooled material that was also used for mRNA sequencing to create two fully congruent paired datasets generated on the same common sources. Each stage x organ/tissue combination is represented as three biological replicates (three separate pools of dissected material of 250–500 seeds each) constituting a sample set.

### Qualitative and quantitative assessment of sequencing and proteomics data

**Fig. 2:**
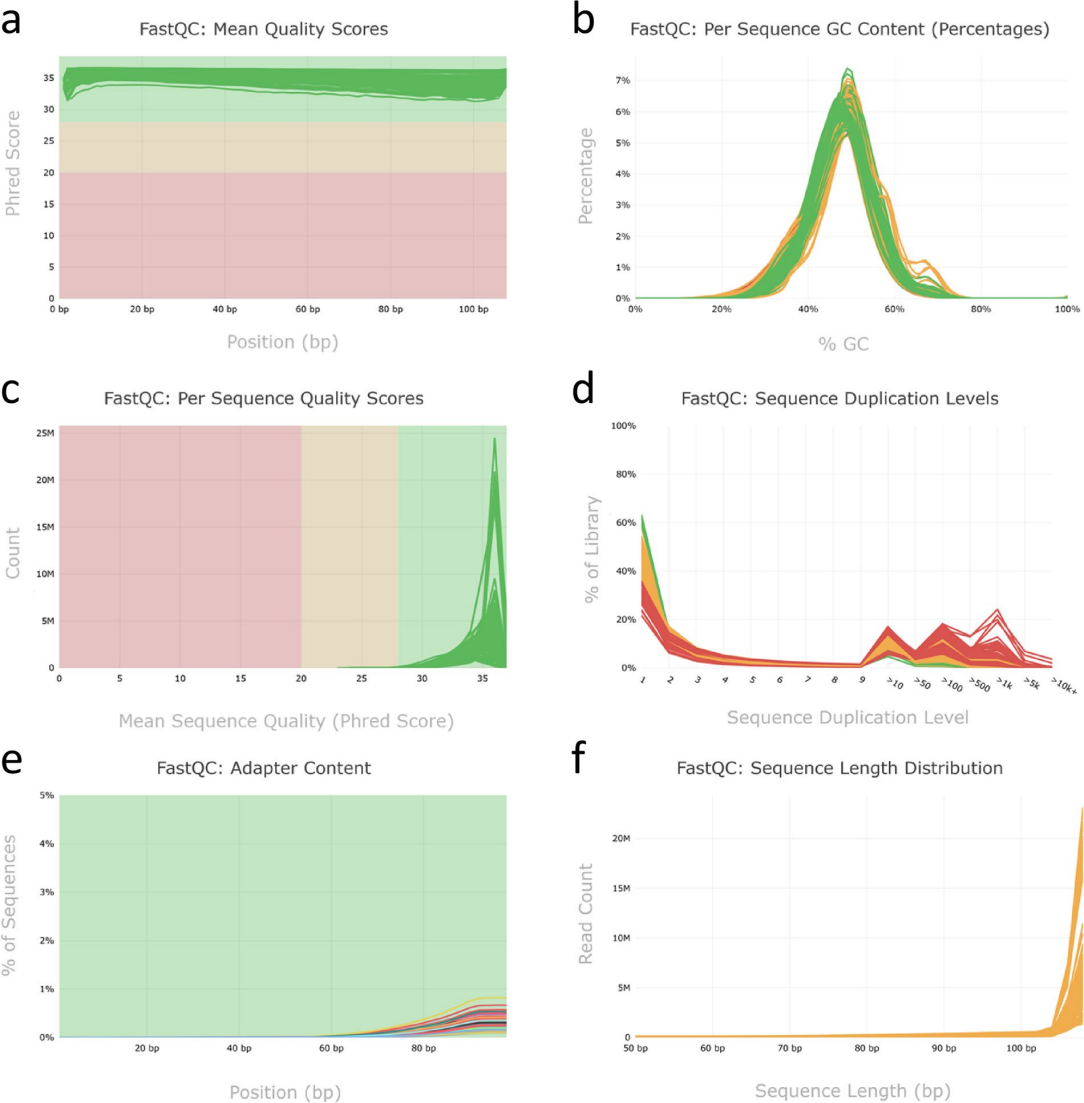
Evaluation of sequence quality after raw read processing. The quality scores of trimmed paired-end RNA-seq reads of all 51 samples were assessed using FastQC (version 0.11.7), and the results were compiled and summarized using MultiQC (version 1.23). Forward (R1) and reverse (R2) reads and different lanes (L001 & L002) were analysed and plotted separately. (**a**) Displays the mean quality scores for each base position across all reads, (**b**) illustrates the per-sequence GC content, **(c)** represents a distribution of the average quality scores per read across all sequences, (**d**) provides information on the sequence duplication levels, **(e)** displays the content of adapter sequences, and (**f**) shows the sequence length distribution.

Samples were sequenced using a S2_XP v1.5 flow cell on a NovaSeq 6000 (Illumina, Berlin, Germany) platform to achieve high coverage of the global transcriptome. To mitigate potential differences related to technical factors, libraries were sequenced in replicates on the two lanes. In total, sequencing resulted in the acquisition of approximately 339.57 gigabases (Gb) pair-end raw data, with an average number of 61 million reads per sample (Table 3). Due to a mix-up of samples during library preparation, five samples needed to be repeated and were sequenced on a separate lane of a further flow cell, hence yielding a higher sequencing depth. The sequencing results were of high quality indicated by an average Phred quality score > 33.9 and an average Q30 value > 83%. To ensure the accuracy and reliability of the data, a series of quality control steps was implemented, including adaptor sequences removal and elimination of low-quality regions of each read. After read trimming, an average of 51 million reads per sample was aligned to the latest *Brassica napus* (Darmor-*bzh* v10)^30^ reference genome version using Hisat2^31^. A meticulous quality assessment of the generated 51 RNA-seq dataset was performed using FastQC^45^ and summarized in a report using MultiQC^46^. QC plots include mean quality scores (Fig. 2a), per sequence GC content (Fig. 2b), per sequence quality scores (Fig. 2c), sequence duplication levels (Fig. 2d), adapter content (Fig. 2e), and sequence length distribution (Fig. 2f). All parameters were in the expected ranges for RNA sequencing data, which establishes a solid foundation for follow-up analyses. The analysis revealed that mean Phred quality scores of all samples are above 30 and that per-sequence quality scores were predominantly concentrated within the range of 30 to 40, indicating a base error rate smaller than 0.1%. The GC distribution of all sequences followed the expected normal distribution. The percentage of clean reads was calculated as 84.2% of the raw sequences (Table 3). The mapping results, represented in Table 3, show that on average 92.4% of the cleaned reads per sample were successfully mapped to the reference genome.

To ensure that performance fluctuations of our LC-MS system did not introduce biases in the dataset during proteomic analysis, all samples were measured in a randomized order^27^. MS raw data were analysed using MaxQuant and Skyline to assess a series of QC parameters which are summarised in Table 4. Additional QC parameters and statistics are provided at figshare ‘04_proteomics_QC_table_extended’^27^. We monitored the chromatographic performance and mass accuracy of the LC-MS system for each run (‘05_proteomics_QC_plots’)^27^. The average chromatographic peak width (full width at half maximum, FWHM) was 4.8 seconds, ranging from 4.6 to 5.1 seconds between all 51 samples^27^, indicating robust chromatography of the peptides. The average mass deviation ranged from 0.1 to 5.2 ppm^27^. To achieve the highest possible peptide mass accuracy across all samples, MaxQuant recalibrates peptide masses for each sample individually, using an initial search with less stringent criteria^38^. After this recalibration, the average mass deviation across all samples ranged from 0.0 to 0.3 ppm^27^, which is well within the set tolerance limit of 10 ppm. Furthermore, the charge distribution of the analysed peptides was compared, revealing a high degree of similarity across all samples, with 72–79% of all peptides being doubly and 19–25% being triply charged^27^.

**Table 4:** Proteomics data quality metrics.

Sample name	Number of protein groups^†^					Mass error [ppm] ^#^	Mean FWMH [s]	TIC area
	Total	Identified by MS/MS	Identified by matching	iBAQ > 0	LFQ > 0			
L401HPKH21P01S1WS	11,440	7,369	4,071	10,671	7,501	0.3	4.9	1.60E + 09
L401HPKH21P02S1WS	11,469	7,606	3,863	10,652	7,568	0.3	4.7	1.62E + 09
L401HPKH21P03S1WS	11,328	7,230	4,098	10,485	7,268	0.2	4.7	1.70E + 09
L401HPKH21P01S2SC	11,370	7,279	4,091	10,560	7,443	0.3	4.7	1.54E + 09
L401HPKH21P02S2SC	11,220	6,823	4,397	10,237	7,162	0.1	4.7	1.57E + 09
L401HPKH21P03S2SC	11,145	6,778	4,367	10,177	7,049	0.1	4.8	1.63E + 09
L401HPKH21P01S2IC	11,234	6,808	4,426	10,283	7,065	0.2	4.9	1.63E + 09
L401HPKH21P02S2IC	11,069	6,590	4,479	10,067	6,832	0.2	5.0	1.69E + 09
L401HPKH21P03S2IC	11,083	6,918	4,165	10,093	6,913	0.2	4.8	1.60E + 09
L401HPKH21P01S2OC	11,241	6,987	4,254	10,408	7,171	0.3	4.7	1.52E + 09
L401HPKH21P02S2OC	11,158	6,605	4,553	10,147	6,908	0.2	4.9	1.70E + 09
L401HPKH21P03S2OC	10,984	6,456	4,528	9,907	6,626	0.3	4.8	1.50E + 09
L401HPKH21P01S2RA	11,309	6,908	4,401	10,527	7,217	0.2	4.8	1.63E + 09
L401HPKH21P02S2RA	10,933	6,477	4,456	9,871	6,672	0.0	4.6	1.33E + 09
L401HPKH21P03S2RA	11,038	6,518	4,520	10,017	6,776	0.2	5.0	1.72E + 09
L401HPKH21P01S3SC	11,251	7,014	4,237	10,325	7,293	0.2	4.8	1.60E + 09
L401HPKH21P02S3SC	11,355	7,403	3,952	10,572	7,467	0.2	4.7	1.54E + 09
L401HPKH21P03S3SC	11,159	7,082	4,077	10,201	7,058	−0.1	4.6	1.47E + 09
L401HPKH21P01S3IC	11,114	6,284	4,830	10,131	6,820	0.3	4.9	1.69E + 09
L401HPKH21P02S3IC	11,015	6,272	4,743	10,031	6,764	0.1	4.9	1.66E + 09
L401HPKH21P03S3IC	11,097	6,814	4,283	10,135	6,927	0.2	4.8	1.61E + 09
L401HPKH21P01S3OC	11,144	6,407	4,737	10,134	6,896	0.2	4.9	1.67E + 09
L401HPKH21P02S3OC	10,825	5,979	4,846	9,604	6,376	−0.1	4.7	1.40E + 09
L401HPKH21P03S3OC	10,986	6,181	4,805	9,903	6,669	0.2	4.9	1.65E + 09
L401HPKH21P01S3RA	10,904	5,605	5,299	9,795	6,470	0.1	5.0	1.71E + 09
L401HPKH21P02S3RA	10,940	5,709	5,231	9,821	6,544	0.1	4.9	1.64E + 09
L401HPKH21P03S3RA	10,957	6,072	4,885	9,888	6,600	0.1	4.9	1.68E + 09
L401HPKH21P01S4SC	11,146	6,737	4,409	10,187	7,073	0.2	4.7	1.55E + 09
L401HPKH21P02S4SC	10,938	6,036	4,902	9,843	6,724	0.2	4.9	1.59E + 09
L401HPKH21P03S4SC	11,073	6,783	4,290	9,990	6,956	0.1	4.8	1.59E + 09
L401HPKH21P01S4IC	10,484	4,470	6,014	9,063	5,901	0.2	5.0	1.58E + 09
L401HPKH21P02S4IC	10,885	5,705	5,180	9,779	6,553	0.1	4.7	1.60E + 09
L401HPKH21P03S4IC	10,684	5,392	5,292	9,346	5,965	0.2	5.0	1.91E + 09
L401HPKH21P01S4OC	10,473	4,279	6,194	8,974	5,779	0.3	5.0	1.57E + 09
L401HPKH21P02S4OC	10,584	5,073	5,511	9,305	6,056	0.2	4.7	1.42E + 09
L401HPKH21P03S4OC	10,565	4,878	5,687	9,230	6,039	0.1	4.8	1.57E + 09
L401HPKH21P01S4RA	10,308	3,967	6,341	8,768	5,567	0.1	5.0	1.58E + 09
L401HPKH21P02S4RA	10,590	4,915	5,675	9,248	6,083	0.0	4.8	1.49E + 09
L401HPKH21P03S4RA	9,983	3,670	6,313	8,233	5,134	0.1	5.1	1.65E + 09
L401HPKH21P01S5SC	10,327	4,598	5,729	8,735	5,676	0.1	4.7	1.37E + 09
L401HPKH21P02S5SC	10,126	4,400	5,726	8,441	5,413	0.0	4.7	1.30E + 09
L401HPKH21P03S5SC	10,543	5,095	5,448	9,140	6,034	0.1	4.9	1.62E + 09
L401HPKH21P01S5IC	9,674	3,392	6,282	7,886	4,902	0.1	4.8	1.49E + 09
L401HPKH21P02S5IC	9,947	3,806	6,141	8,265	5,063	0.0	5.1	1.82E + 09
L401HPKH21P03S5IC	9,645	3,487	6,158	7,820	4,868	0.0	4.8	1.45E + 09
L401HPKH21P01S5OC	9,867	3,526	6,341	8,134	5,101	0.3	4.8	1.59E + 09
L401HPKH21P02S5OC	9,608	3,709	5,899	7,870	4,866	0.0	4.7	1.29E + 09
L401HPKH21P03S5OC	9,427	3,563	5,864	7,603	4,608	0.2	4.7	1.33E + 09
L401HPKH21P01S5RA	9,849	3,505	6,344	8,175	5,143	0.1	4.9	1.59E + 09
L401HPKH21P02S5RA	9,948	4,026	5,922	8,343	5,203	0.0	4.7	1.52E + 09
L401HPKH21P03S5RA	9,732	4,034	5,698	8,041	5,069	0.2	4.7	1.43E + 09

^†^ Filtered for non-plant contaminants and reversed sequences and proteins only identified based on modified peptides

^#^mean mass deviation of peptides across all samples after internal recalibration

abbreviations: TIC = total ion chromatogram; FWHM = full width at half maximum (chromatographic peak width)

Note: additional QC parameters and statistics are provided at figshare ‘04_proteomics_QC_table_extended’^27^.

Depending on the sample, 31,826 to 57,215 peptides and 9,427 to 11,469 protein groups were identified per sample. On average, 38% of all protein groups were covered with 2 or more unique peptides. A large portion of all proteins in each sample was covered with only one unique peptide. The relatively low number of unique peptides can be explained by the high similarity of numerous homologous genes of the A and C subgenomes, from which correspondingly similar or identical protein sequences were derived for our proteome analysis. Thus, approximately 50% of all identified peptides could not be uniquely assigned to a single protein based on the annotated genome sequence. Consequently, many proteins that were assigned to only one unique peptide also had multiple razor peptides associated with them. 7,603 to 10,671 proteins could be quantified using iBAQ and 4,608 to 7,568 proteins using the more stringent MaxLFQ strategy. Detailed information on the identification and quantification rates of all samples is summarized at figshare (‘04_proteomics_QC_table_extended’)^27^.

Notably, there is a tendency towards a higher average sequence coverage and higher peptide and protein identifications in samples of the earlier compared to later developmental stages. This trend continues in the number of quantifiable proteins per sample. The substantial changes in protein composition within the samples are responsible for this effect. During seed maturation, increasing amounts of various storage proteins are produced, which make up a very substantial portion (up to 85–90%) of the total protein content by the end of maturation^52–54^. This extreme abundance of storage proteins complicates the detection of other proteins.

To ensure that the high sample dynamics did not affect our assessment of system performance, commercial HeLa peptide standards were additionally measured and evaluated as QC samples at regular intervals. Additional information and plots are provided at figshare (‘06_HeLa_QC_standards’ & ‘07_HeLa_QC_plots’)^27^. All the verified parameters indicated robust system performance.

### Transcriptomic and proteomic coverage and sample distribution

**Fig. 3:**
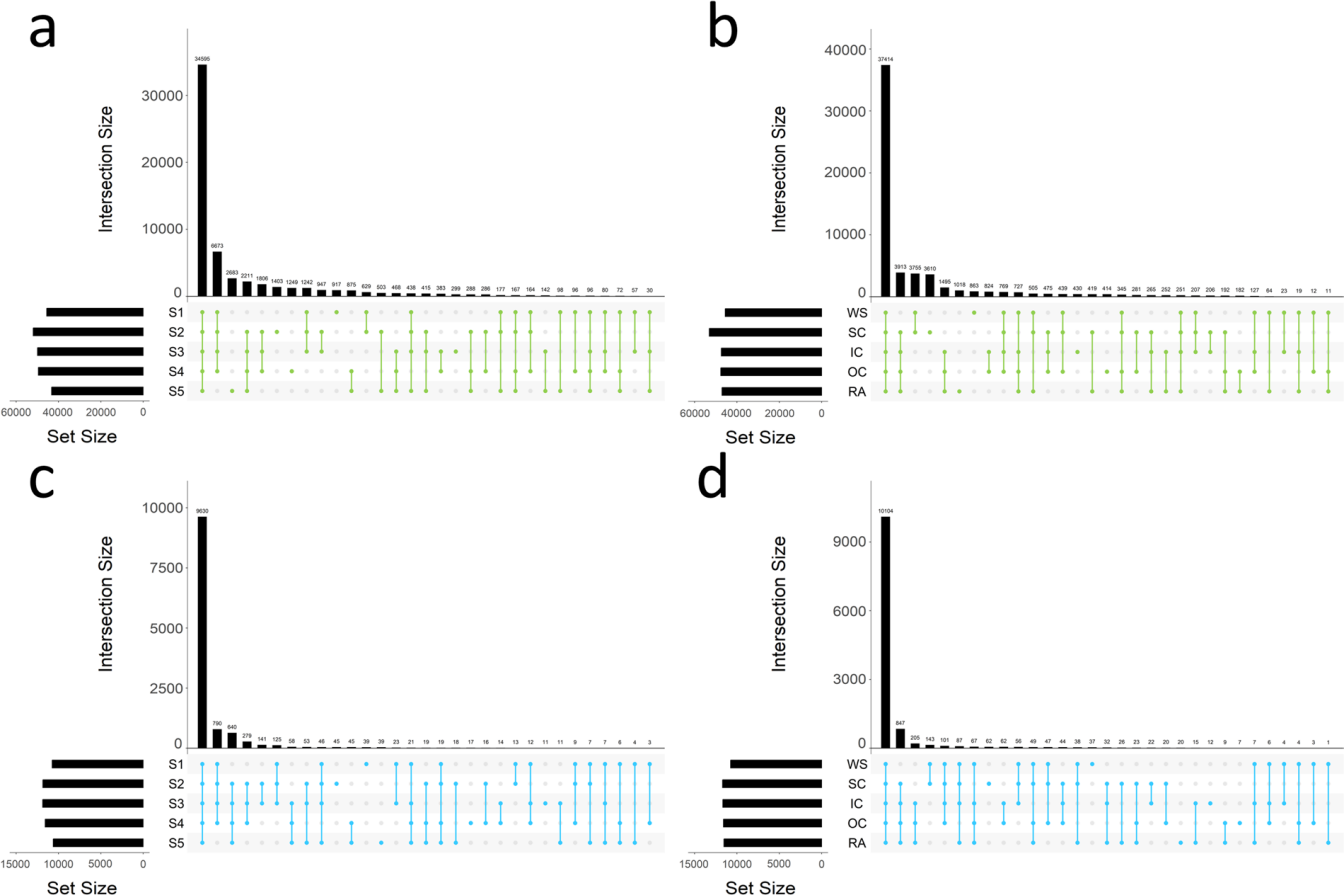
Protein groups and transcripts detected across stages and organs. The intersections of detected transcripts and proteins across sample sets were visualized using UpSet plots. Panels (**a** and **b**) display the transcriptome dataset (tpm; green colour) and (**c** and **d**) the proteome data (iBAQ values; blue colour), for the five developmental stages (S1 to S5) and individual organs/tissues (WS = whole seed; IC = inner cotyledon; OC = outer cotyledon; and RA = radicle), respectively. Transcripts and proteins were filtered for low expressed features: ≥10 raw counts and ≥1 tpm for transcripts and iBAQ values ≥ 1 for proteins, detected in at least two of three biological replicates, respectively.

The latest Darmor-*bzh* genome version (v10)^30^ used for sequence mapping is a widely recognized reference resource in rapeseed genome research, encompassing transcript information for 108,190 protein-coding genes of the two (A & C) subgenomes. To normalize the transcript quantities of each coding gene, the mapping results were normalized using tpm (transcripts per million). Using the workflow described above, we quantified the mRNA expression of 57,273 genes (53% of the genome), with ≥ 10 raw counts and ≥1 tpm in at least two of three biological replicates of at least one sample set (one sample set consists of the three biological replicates of a certain stage x organ/tissue combination). The number of expressed genes ranged from 27,681 detected in the radicle of stage 4 to 45,300 in the whole seed samples of stage 1 (Table 2). A core subset of genes (n = 18,184) was detected as expressed in all 17 stage and organ/tissue combinations. In addition, we identified a total 84,376 peptide sequences corresponding to 12,200 protein groups by LC-IMS-MS/MS. 5,931 protein groups contained unambiguous single proteins, 3,636 contained two proteins, which often but not exclusively represent the homeologs of the two subgenomes and 2,633 protein groups were ambiguously associated with more than two proteins. Grouping samples by developmental stages, the highest intersection was found for genes expressed across all five stages (n = 34,595), followed by genes with shared expression across the first four developmental stages (n = 6,673), and genes unique to the maturation stage (n = 2,683; Fig. 3a). Regarding organs/tissues, by far the highest intersection was detected across all five organs/tissues (n = 37,414), followed by organs/tissues excluding whole undissected seeds (n = 3,913), and the overlap of genes only expressed in whole seeds and the seed coat (n = 3,755; Fig. 3b). The overall distribution of protein abundances (iBAQ) displayed very similar patterns compared to the transcripts. The highest intersections of protein groups were again observed across all five developmental stages (n = 9,630; Fig. 3c) and across all organs/tissues (n = 10,104; Fig. 3d). Notably, in later stages, lower numbers of peptides/proteins were detected (Table 2). For follow-up analyses, we provide both, iBAQ values as proxies to determine the detection of a protein in each sample, as well as normalized LFQ intensities for relative quantification across samples. LFQ intensities > 0 were reported by MaxQuant for 9,643 of the 12,200 protein groups.

### Reproducibility and congruence of the datasets

**Fig. 4:**
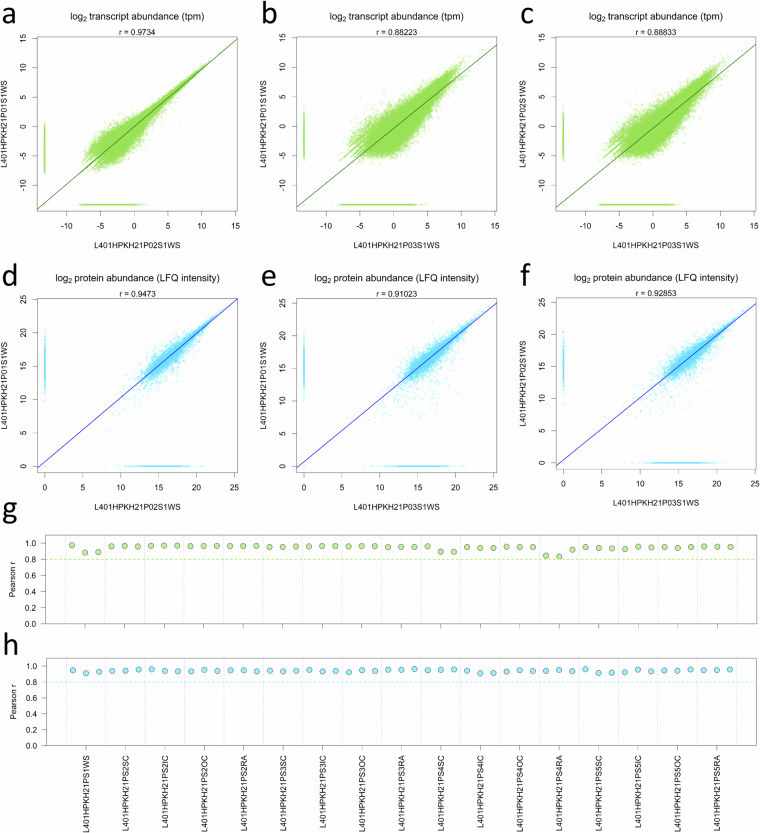
Correlation between biological replicates of transcriptome and proteome data. Subfigures (**a** to **c**) display pairwise correlation for the three biological replicates of the transcriptome (tpm values; green colour) and (**d** to **f**) for the proteome data (LFQ intensities, blue colour), exemplarily for the first sample set (L401HPKH21PS1W = whole seeds; stage 1 ‘pre-storage’). Subfigures, (**g** and **h)** display the Person correlation coefficient for all 17 sample sets (stage and tissue/organ combinations). Values of zero were displayed in the plots, but treated as missing for correlation analysis. The three datapoints per group indicate the comparisons between replicates in the following order: replicate 1 vs. 2, 1 vs. 3, and 2 vs. 3, respectively.

**Fig. 5:**
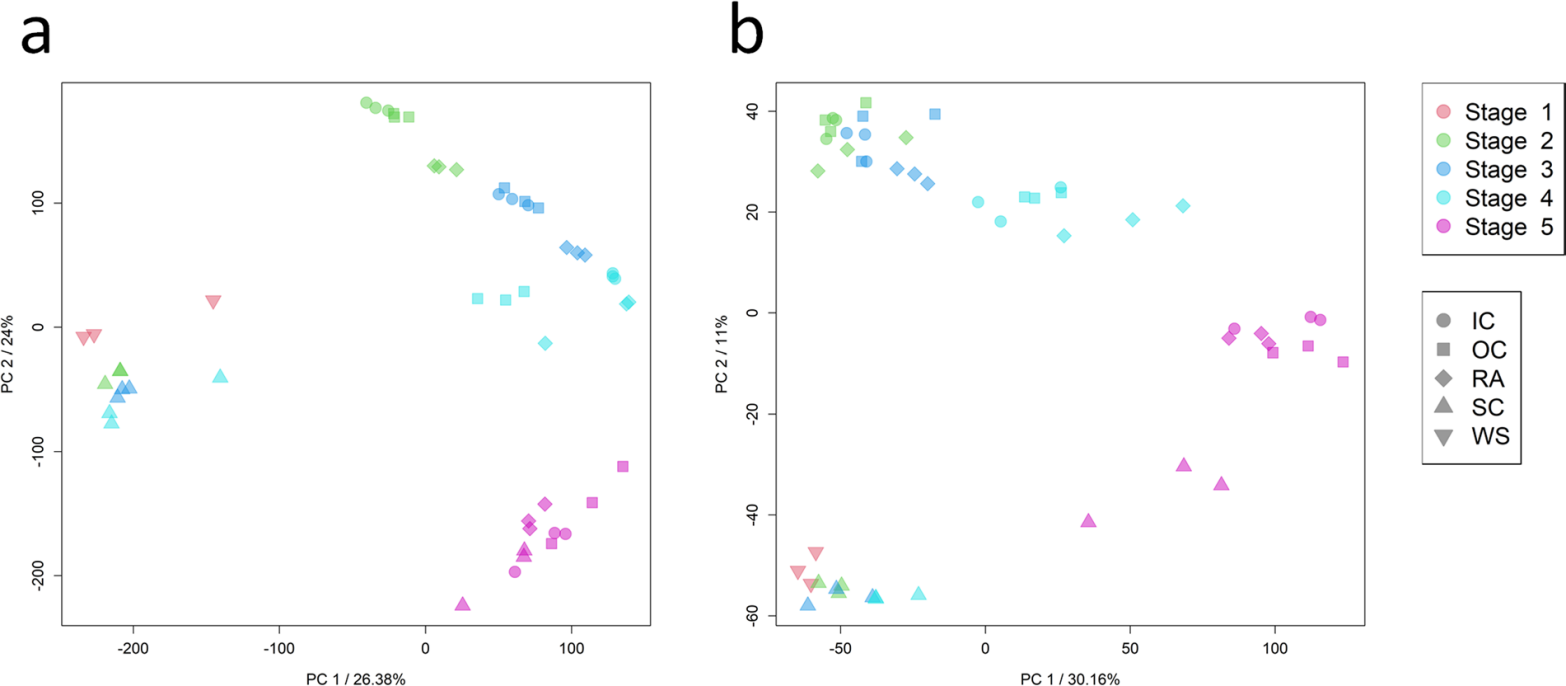
Principal component analysis of the transcriptome and proteome data. Principal component analyses for (**a**) transcriptome data (filtered tpm; n = 57,273), (**b**) proteome data (iBAQ values, n = 12,200). Both datasets were scaled, centred, and the proteome data were log_2_ transformed before the calculation. Principal components (PCs) were calculated using singular value decomposition and the first two PCs are plotted. The axes indicate the percentage of explained variance. Developmental stages are represented by colours and the tissues by symbols, respectively.

**Fig. 6:**
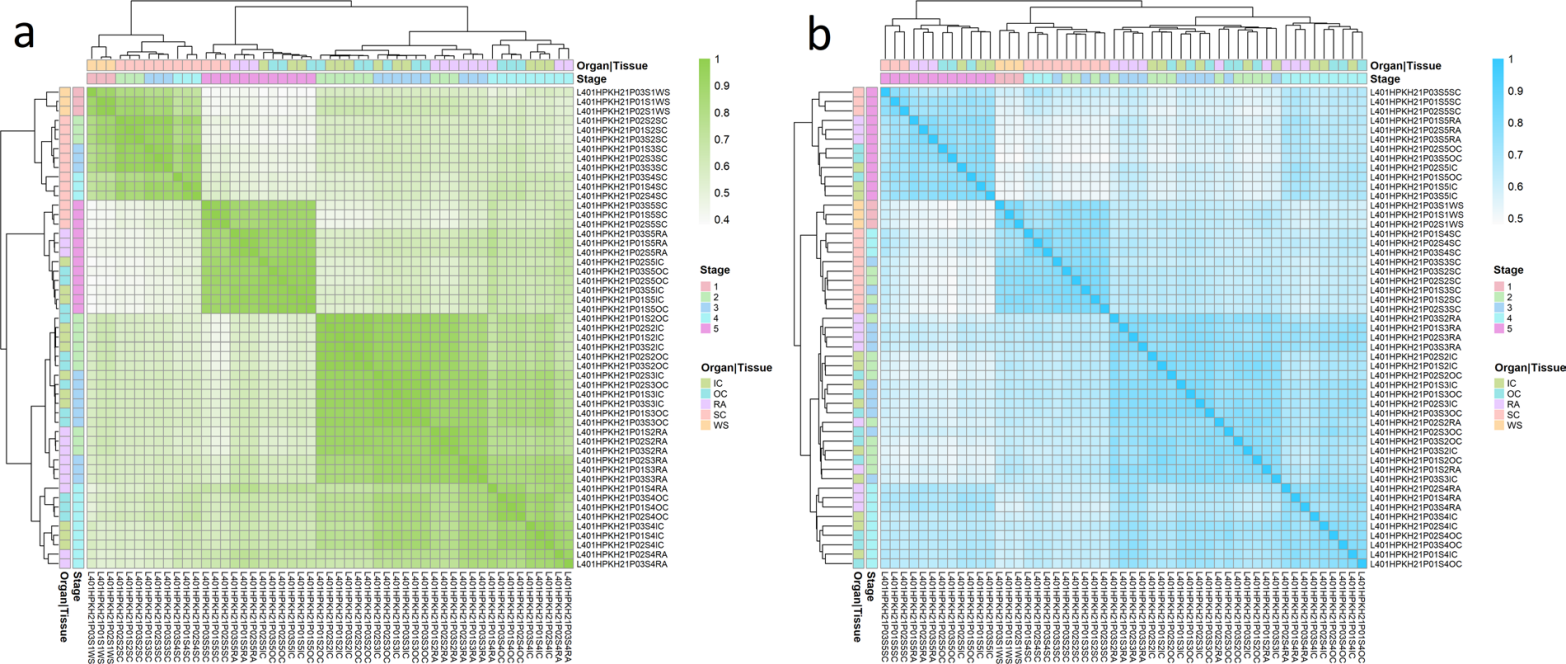
Pairwise correlation matrices and hierarchical clustering. Pearson correlation matrices computed across all 51 samples for (**a**) transcriptome data (green colour; filtered tpm; n = 57,273), (**b**) proteome data (blue colour; LFQ intensity values, n = 12,200). Both datasets were log_2_ transformed after addition of a pseudo count (0.1 tpm or 1e-5 for LFQ intensity, respectively). Both correlation matrices were converted to distance matrices (1 - cor) and a hierarchical cluster analysis was performed. The dendrograms are shown at the top and left sites alongside a colour coding indicating developmental stages and organ/tissue, respectively.

Subsequently, correlation analyses between the three biological replicates for each sample set were conducted that indicate little variation and strong similarity among the replicates with Pearson correlation coefficients above 0.8 in all cases (Fig. 4). Overall, this demonstrates a high level of reproducibility. Certain transcriptome samples, such as whole seed stage 1, seed coat stage 4, and radicle stage 4, exhibit slightly higher variability when compared to the proteome data, which show a more uniform pattern. The transcriptional differences between samples were evaluated by a principal component analysis (PCA) using the tpm values. The results demonstrate a separation of samples by developmental stages and organs (Fig. 5a). While the second to fourth stage are morphologically more similar and characterized by the growth of the embryo, the fifth stage is characterized by the distinct changes due to maturation and desiccation. This is also reflected in the PCA. Notably, three larger sub-clusters can be observed in the biplot of the first two principal components (PCs), explaining 26.38% and 24% of the variance, respectively: cluster one formed by the samples of the whole seeds from stage 1 and the seed coat samples of stages 2 to 4; cluster two with embryonic green tissue from stages 2 to 4; and a distinct third cluster comprising all tissues of stage 5. Biological replicates mainly cluster close together with minor variation. The PCA of the proteome (iBAQ) data revealed very similar patterns as observed for the transcriptome (Fig. 5b). It is noteworthy that biological replicates display a tendency to cluster together, further underlining the high degree of reproducibility achieved. These results show a certain degree of variability within sample sets as expected for biological replicates. Variation between the sample sets is driven by stages followed by organs/tissues at both omics levels. Similar to the PCA analysis, the pairwise correlation matrices calculated across all 51 samples indicate a high congruence between the transcriptome and proteome datasets (Fig. 6), highlighting three major clusters of samples. The hierarchical clustering shows that most biological replicates cluster closely together, in particular for the whole seed, seed coat, and most for the radicle samples, with a clear distinction between the maturation stage (S5) and all earlier stages. The biological replicates for the inner and outer cotyledons for stages 2 and 3 are more similar to each other and form a slightly less distinct larger cluster of embryonic tissues.

## Usage Notes

In this study, we generated a comprehensive omics dataset covering a series of five key stages of seed development and up to four different dissected seed organs/tissues using mRNA-sequencing and LC-IMS-MS/MS-based proteomics. For the transcriptomic data, we have included the raw dataset available in fastq format, as well as the processed data presented as sorted and indexed bam files, and in csv format. The processed data consists of both, raw count and normalized expression (tpm) values. Proteomics data are available as raw mass spectrometric files, as well as quantitative data within samples (iBAQ) and normalized between samples (LFQ intensities). With the following examples, we intend to illustrate how this dataset, with its multifactorial design, might be exploited through different approaches: (1) The mRNA-seq and proteome datasets can be used to examine the variation in expression profiles, and to identify differentially expressed genes (DEGs) and proteins (DEPs), between tissues at particular stages and across the developmental time series, using DESeq 2^55^, edgeR^56^, limma-voom^57^, DEP2^58^ or similar analysis pipelines. This approach may also be useful for investigating unknown common factors associated with various developmental processes, storage accumulation and maturation. (2) Additionally, our findings provide useful data for research focused on clustering of the data by hierarchical or k-means algorithms, providing insights into the specific genes and sample combinations that exhibit notable differential expression patterns. (3) The identification of tissue- and stage-specific gene expression and protein abundance might help in the generation of expression markers. (4) The results of the proteomics and transcriptomics layers can further be integrated on a correlative basis, addressing the overlap of DEGs and differentially abundant proteins (DEPs), or provide the basis or a validation set for transcriptomic and regulatory networks. (5) Another level of analysis could address the correlations of mRNA-protein pairs with the opportunity to discover instances of post-transcriptional regulation. However, this might prove challenging due to the allotetraploid nature of the *Brassica* *napus* genome and because only a subset of the transcript / protein pairs are unambiguous. (6) Finally, due to the deep coverage of the proteome and the temporal and spatial diversity of the samples, covering distinct stages of seed development, the data provide a valuable addition to a future *Brassica napus* proteome atlas in reference to the Arabidopsis^59^ or maize^60^ peptide atlas.

Moreover, this dataset establishes a foundational reference that can be leveraged for comparative analyses with future datasets. By integrating data from other genotypes and/or examining seeds formed under various environmental stresses, researchers may gain deeper insights into adaptive and stress-responsive mechanisms. Such comparisons could reveal genotype-specific variation and stress-induced changes in gene expression and protein abundance, offering a valuable resource for advancing our understanding of seed development and resilience in *Brassica napus* and related species.

## Data Availability

Data analysis procedures have been described in detail in the Methods section. The following software was used in the study and run with default parameters if not stated otherwise: R https://www.r-project.org/ (version 4.3.1 for Windows) RStudio https://posit.co/download/rstudio-desktop/ (version 2023.12.1 Build 402) FastQC https://www.bioinformatics.babraham.ac.uk/projects/fastqc (version 0.11.7) MultiQC https://github.com/MultiQC/MultiQC (version 1.23) Fastp https://github.com/OpenGene/fastp?tab=readme-ov-file (version 0.20.0) Hisat2 https://daehwankimlab.github.io/hisat2/download (version 2.1.0) HTSeq https://pypi.org/project/HTSeq (version 1.99.2) MaxQuant https://www.maxquant.org/ (version 2.0.3.0) Skyline https://skyline.ms/project/home/software/Skyline/begin.view (version 24.1.0.414)Instant Clue http://www.instantclue.uni-koeln.de/ (version 0.12.2) Principal component analysis: R package pcaMethods (version 1.92.0) UpSet plots: R package UpSetR (version 1.4.0) Heatmaps: R package pheatmap (version 1.0.12) *Brassica napus* Darmor-bzh reference genome (v10): https://www.genoscope.cns.fr/externe/plants/chromosomes.html
